# Developmental Exposure of Rats to Chlorpyrifos Elicits Sex-Selective Hyperlipidemia and Hyperinsulinemia in Adulthood

**DOI:** 10.1289/ehp.8133

**Published:** 2005-06-02

**Authors:** Theodore A. Slotkin, Kathleen K. Brown, Frederic J. Seidler

**Affiliations:** 1 Department of Pharmacology and Cancer Biology, Duke University Medical Center, Durham, North Carolina, USA; 2 GlaxoSmithKline Inc., Research Triangle Park, North Carolina, USA

**Keywords:** Barker hypothesis, chlorpyrifos, cholesterol, diabetes mellitus type 2, insulin, organophosphates, triglycerides

## Abstract

Developmental exposure to chlorpyrifos alters cell signaling both in the brain and in peripheral tissues, affecting the responses to a variety of neurotransmitters and hormones. We administered 1 mg/kg/day chlorpyrifos to rats on postnatal days 1–4, a regimen below the threshold for systemic toxicity. When tested in adulthood, chlorpyrifos-exposed animals displayed elevations in plasma cholesterol and triglycerides, without underlying alterations in nonesterified free fatty acids and glycerol. This effect was restricted to males. Similarly, in the postprandial state, male rats showed hyperinsulinemia in the face of normal circulating glucose levels but demonstrated appropriate reduction of circulating insulin concentrations after fasting. These outcomes and sex selectivity resemble earlier findings at the cellular level, which identified hepatic hyperresponsiveness to gluconeogenic inputs from β-adrenoceptors or glucagon receptors. Our results thus indicate that apparently subtoxic neonatal chlorpyrifos exposure, devoid of effects on viability or growth but within the parameters of human fetal or neonatal exposures, produce a metabolic pattern for plasma lipids and insulin that resembles the major adult risk factors for atherosclerosis and type 2 diabetes mellitus.

Chlorpyrifos, one of the most widely used organophosphorus pesticides, is increasingly restricted in the United States because of its adverse effects on fetal and neonatal brain development [Barone et al. 2000; Landrigan 2001; Slotkin 2004; U.S. Environmental Protection Agency (EPA) 2000, 2002]. Nevertheless, organophosphates, including chlorpyrifos, still account for up to 50% of all insecticide application worldwide (Casida and Quistad 2004). Although the systemic toxicity of these pesticides resides in their ability to inhibit cholinesterase, other mechanisms contribute to their developmental neurotoxicity, notably the targeting of cell signaling cascades governing neuronal and hormonal signals that are essential to cell differentiation and homeostatic regulation (Barone et al. 2000; Casida and Quistad 2004; Gupta 2004; Pope 1999; Schuh et al. 2002; Slotkin 2004). Of these, the pathway synthesizing cyclic AMP (cAMP), controlled by adenylyl cyclase, appears to be among the most prominent sites for disruption by chlorpyrifos (Meyer et al. 2003, 2004a, 2004b; Olivier et al. 2001; Ward and Mundy 1996; Zhang et al. 2002).

Adenylyl cyclase and cAMP also participate in important metabolic, cardiovascular, and hormonal functions in the periphery, and we recently found that neonatal chlorpyrifos exposure leads to disruption of cardiac and hepatic cell signaling in adulthood (Meyer et al. 2004b). Perhaps most critically, chlorpyrifos-exposed males showed hyperreactivity of hepatic adenylyl cyclase to stimulation of β-adrenoceptors or glucagon receptors, inputs that are responsible for promoting gluconeogenesis and the consequent rise in circulating glucose levels. If these cellular alterations do indeed result in changes in hepatic function and responsiveness, then neonatal chlorpyrifos exposure might be expected to elicit long-term hyperglycemia and associated metabolic abnormalities, or alternatively, insulin hypersecretion might be required to offset the promotion of gluconeogenic signals. In the present study, we demonstrate that male rats exposed neonatally to chlorpyrifos display hyperinsulinemia and hyperlipidemia in adulthood, two of the major risk factors for type 2 diabetes mellitus and atherosclerosis. Our working hypothesis thus complements the Barker hypothesis, which draws a connection between low birth weight and the subsequent risk of coronary artery disease and diabetes (Barker 2003; Phillips 2002), extending the same outcomes into the realm of exposure to environmental toxicants even in the absence of growth retardation.

## Materials and Methods

### Animal treatments.

All experiments were carried out in accordance with the *Guide for the Care and Use of Laboratory Animals* as adopted and promulgated by the National Institutes of Health (Institute of Laboratory Animal Resources 1996). Timed-pregnant Sprague–Dawley rats (Charles River, Raleigh, NC) were housed in breeding cages, with a 12/12 hr light/dark cycle and free access to food and water. On the day of birth, all pups were randomized and redistributed to the dams with a litter size of 10 to maintain a standard nutritional status. Randomization within the respective treatment groups was repeated at intervals of several days, and in addition, dams were rotated among litters to distribute any maternal caretaking differences randomly across litters and treatment groups. Chlorpyrifos (Chem Service, West Chester, PA) was dissolved in dimethyl sulfoxide to provide consistent absorption (Whitney et al. 1995) and was injected subcutaneously at a dose of 1 mg/kg in a volume of 1 mL/kg once daily on postnatal days 1–4; control animals received equivalent injections of the dimethyl sulfoxide vehicle. This regimen has been shown previously to produce developmental neurotoxicity without eliciting growth retardation or any other signs of systemic toxicity (Aldridge et al. 2004; Meyer et al. 2004a, 2004b; Slotkin 2004; Song et al. 1997; Whitney et al. 1995). Indeed, neonatal brain cholinesterase inhibition is only about 25% (Song et al. 1997), well below the 70% threshold necessary for symptoms of cholinergic hyperstimulation (Clegg and van Gemert 1999), thus resembling the nonsymptomatic exposures reported in pregnant women (De Peyster et al. 1993). Moreover, the dose used here is well within the range of typical fetal and childhood exposures after routine application (Gurunathan et al. 1998; Ostrea et al. 2002).

Animals were weaned on postnatal day 21. Tests were performed using eight rats per sex per treatment group, with no more than one male and one female from each litter. Starting immediately after weaning, animals were handled every few days to accustom them to removal from the cage and contact with the investigators. At 110 days of age, during the active (dark) cycle, animals were placed in a Plexiglas restrainer, and two blood samples (total volume, 500 μL) were obtained from each animal by tail vein venipuncture, using a 23-gauge butterfly, one without added anticoagulant and the other containing EDTA. Handling was then maintained for the ensuing 10 days, at which time animals were fasted 8 hr, and a second pair of samples were obtained. Sera were analyzed using an Olympus Au640 Clinical Chemistry Analyzer (Olympus America Inc., Melville, NY), and insulin was determined using a commercial radioimmunoassay kit (Linco Research, St. Charles, MO). Samples containing EDTA were analyzed for total and glycosylated hemoglobin using a ColumnMate Analyzer (Helena Laboratories, Beaumont, TX). At no time during the restraint or venipuncture did the animals show overt signs of distress such as struggling or vocalizations.

### Data analysis.

Data are presented as means and standard errors. To establish the effects of chlorpyrifos and its relationship to other variables, a multivariate analysis of variance (ANOVA; data log-transformed because of heterogeneous variance) was first conducted, encompassing neonatal treatment, sex, and feeding status (nonfasted vs. fasted, treated as a repeated measure, because each animal was evaluated sequentially for both states). Where chlorpyrifos treatment interacted with the other variables, data were then subdivided to permit lower-order ANOVAs, followed where appropriate by Fisher’s protected least significant difference to identify individual values for which the chlorpyrifos groups differed from the corresponding control; however, in the absence of interactions, only main chlorpyrifos treatment effects are reported. For all tests, significance was assumed at *p* < 0.05.

## Results and Discussion

**Figure 1:**
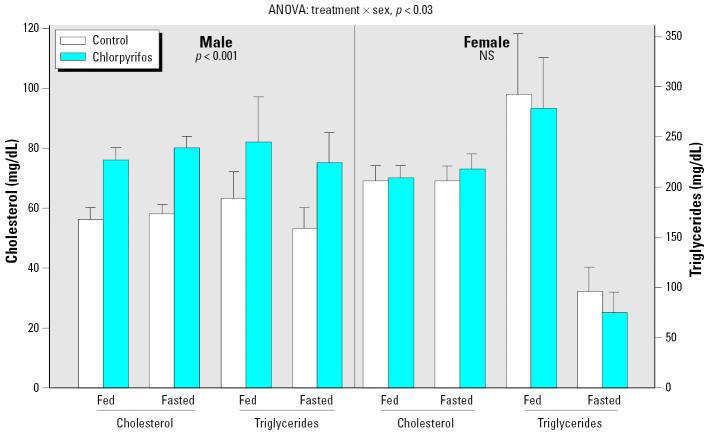
Effects of neonatal chlorpyrifos exposure on plasma lipids in adulthood. NS, not significant. Values shown are mean ± SE. ANOVA across all measures, feeding status, and sex appears at the top; data were subdivided into males and females because of the significant treatment × sex interaction. The main chlorpyrifos treatment effect for each sex is shown within the panel; separate lower-order tests for cholesterol or triglycerides in fed and fasted states were not carried out because of the absence of interactions of treatment × feeding status or treatment × lipid subtype. Note the different scales for cholesterol and triglycerides.

Neonatal chlorpyrifos exposure had no effect on growth or viability (data not shown), or on body weights of male or female rats in adulthood, nor were there any significant alterations in plasma levels of nonesterified free fatty acids or glycerol (Table 1). Nevertheless, cholesterol and triglycerides displayed significant elevations that were distinctly sex selective (treatment × sex interaction, *p* < 0.03), with a preferential effect in males (Figure 1). Both cholesterol and triglycerides were increased by about 35%, an effect that persisted even when the animals were fasted.

**Figure 2:**
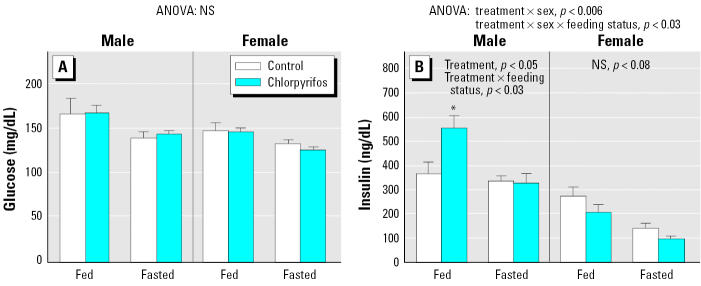
Effects of neonatal chlorpyrifos exposure on plasma glucose (*A*) and insulin (*B*) in adulthood. NS, not significant. Values shown are mean ± SE. ANOVA across feeding status and sex appears at the top of each panel. For insulin levels, lower-order analyses were run separately for males and females and for fed and fasted states because of the interaction of treatment with both feeding status and sex. Across both treatment groups, glucose and insulin levels were significantly lower in females than in males (main effect of sex, *p* < 0.002 for glucose, *p* < 0.0001 for insulin) and were reduced by fasting (main effect of feeding status, *p* < 0.0003 and *p* < 0.0001, respectively). *Significantly different from the corresponding control.

In contrast to the robust effect on plasma lipids, glucose concentrations in chlorpyrifos-exposed animals remained entirely within the normal range for either males or females (Figure 2A), nor did we see any change in the percentage of glycosylated hemoglobin (data not shown). The chlorpyrifos group also showed the typical reduction in glucose levels when fasted and values for females were lower than for males, just as in the control group. Nevertheless, the concentration of insulin was markedly elevated in nonfasted male rats, > 60% higher than in controls (Figure 2B). Fasting restored the insulin level to normal. In contrast, female rats exposed to chlorpyrifos did not show an elevation in insulin and actually showed a slight decrease at the margin of statistical significance. The normal sex differences in these measures as seen in the control groups reproduce those reported previously (O’Regan et al. 2004).

Our results thus indicate that apparently subtoxic neonatal chlorpyrifos exposure, devoid of effects on viability or growth and within the parameters of human fetal or neonatal exposures, produces a metabolic pattern for plasma lipids and insulin that resembles the known major risk factors and predictors for the appearance of atherosclerosis and type 2 diabetes mellitus in adulthood (Davis and Edelman 2004; Reaven et al. 2004). The sex selectivity and response to fasting both point to specific mechanisms underlying these effects. In our earlier work, we found that neonatal chlorpyrifos exposure produces hyperresponsiveness of hepatic cell signaling mediated through adenylyl cyclase, an effect specific to males (Meyer et al. 2004b). The promotional effect extends to two receptors linked to gluconeogenesis, the β-adrenoceptor and the glucagon receptor, and accordingly, it might be expected that these animals would show hyperglycemia. However, as shown here, circulating glucose levels remain essentially normal but only because the effects on gluconeogenic signals are offset by a sustained elevation in post-prandial circulating insulin concentrations. This counterbalanced relationship is reinforced by the fact that fasting restored insulin levels to normal: the feedback regulation of insulin release in response to reduced glucose availability during fasting is intact in the chlorpyrifos group. Under normal dietary conditions, the chlorpyrifos-exposed animals thus display hyperinsulinemia, a characteristic of the prediabetic state in type 2 diabetes mellitus, particularly in obese individuals (Davis and Edelman 2004; Reaven et al. 2004); however, in this case, the hyperinsulinemia exists even in animals with a normal body weight.

Just as found for insulin, male rats exposed to neonatal chlorpyrifos showed elevated plasma cholesterol and triglycerides in adulthood, thus sharing one of the major risk factors for human atherosclerosis. Furthermore, elevated postprandial triglycerides are yet another component of the metabolic syndrome that confers a significant risk of vascular disease. In the present study, there was no evidence of increased *in vivo* lipolysis (i.e., insulin resistance at the level of the adipocyte) because nonesterified fatty acids and glycerol were within normal limits. Unlike the situation for insulin, fasting did not reverse the effects on plasma cholesterol and triglycerides, likely reflecting their longer biologic half-life. Again, the main finding is that an otherwise subtoxic developmental exposure to chlorpyrifos leads to hyperlipidemia similar to that found in individuals prone to cardiovascular disease.

The present results point to a distinct sex disparity in the metabolic consequences of neonatal chlorpyrifos exposure, findings in keeping with effects on hepatic cell signaling as reported previously (Meyer et al. 2004b). Indeed, many other aspects of the developmental toxicity of chlorpyrifos similarly display sex differences, including neurochemical and behavioral effects (Aldridge et al. 2004, 2005; Levin et al. 2001; Moser et al. 1998). The specific mechanisms underlying the targeting of males or females is not yet known, but it is important to note that the impact on brain development targets both males and females, albeit with different patterns of effects. In contrast, the present results indicate metabolic effects restricted to males, so it is unlikely that the underlying mechanisms are the same as those involved in the neurobehavioral effects of chlorpyrifos. In keeping with the present findings, many other developmental disruptors similarly produce sexually dimorphic changes in cardiovascular and/or metabolic outcomes in males and females, both in animal studies and in humans (Adair and Cole 2003; Khan et al. 2003; O’Regan et al. 2004). Taken together with our findings, sex differences clearly need to be taken into account in future evaluations of similar outcomes from developmental chlorpyrifos exposure.

In its original formulation, the Barker hypothesis related prenatal factors leading to reduced birth weight with the subsequent development of cardiovascular disease and diabetes (Barker 2003). The present results point to similar outcomes evoked by an apparently “safe” exposure to a common environmental toxicant, even when normal growth parameters are maintained. In conjunction with our earlier findings for effects of neonatal chlorpyrifos exposure on hepatic cell signaling related to metabolic function (Meyer et al. 2004b), we can now provide a mechanistic link between cellular events and the appearance of hyperinsulinemia and hyperlipidemia in adulthood. Of course, because atherosclerosis and type 2 diabetes are multifactorial diseases, it is unlikely that chlorpyrifos exposure by itself would elicit these disease outcomes; nevertheless, the fact that chlorpyrifos evokes two of the metabolic changes most highly associated with these diseases implies that such exposures may increase risk or vulnerability to other contributory components. The standard view of chlorpyrifos, and potentially other organophosphorus insecticides, as developmental toxicants that specifically target the nervous system may thus require substantial revision.

## Figures and Tables

**Table 1:** Body weights, nonesterified free fatty acids, and glycerol.

	Male		Female	
Measure, age, and feeding status	Control	Chlorpyrifos	Control	Chlorpyrifos
Body weight (g)				
110 days, fed	539 ± 19	547 ± 19	301 ± 7	298 ± 9
120 days, fasted	548 ± 16	568 ± 20	317 ± 6	302 ± 9
Nonesterified free fatty acids (μEq/dL)				
110 days, fed	70 ± 14	67 ± 11	60 ± 8	87 ± 14
120 days, fasted	116 ± 9	112 ± 9	121 ± 11	130 ± 8
Glycerol (mg/dL)				
110 days, fed	24 ± 3	22 ± 3	26 ± 2	31 ± 3
120 days, fasted	28 ± 2	25 ± 1	31 ± 1	27 ± 1

μEq, microequivalents. Values shown are mean ± SE. ANOVA for each set of measures indicates no significant treatment differences. By itself, fasting increased nonesterified free fatty acids (main effect of feeding status, *p* < 0.0001); glycerol values in females were significantly higher overall than in males (main effect of sex, *p* < 0.03).
